# Myxozoan Adhesion and Virulence: *Ceratonova shasta* on the Move

**DOI:** 10.3390/microorganisms7100397

**Published:** 2019-09-26

**Authors:** Gema Alama-Bermejo, Astrid S. Holzer, Jerri L. Bartholomew

**Affiliations:** 1 Department of Microbiology, Oregon State University, Corvallis, OR 97331, USA; barthoje@oregonstate.edu; 2 Centro de Investigación Aplicada y Transferencia Tecnológica en Recursos Marinos Almirante Storni (CIMAS–CCT CONICET–CENPAT), 8520 San Antonio Oeste, Argentina; 3 Institute of Parasitology, Biology Centre, Czech Academy of Sciences, 37005 České Budějovice, Czech Republic; astrid.holzer@paru.cas.cz

**Keywords:** blebbing, cell protrusion, myxozoan adhesion, rainbow trout, motility factors, integrin beta

## Abstract

Motility factors are fundamental for parasite invasion, migration, proliferation and immune evasion and thus can influence parasitic disease pathogenesis and virulence. Salmonid enteronecrosis is caused by a myxozoan (Phylum Cnidarian) parasite, *Ceratonova shasta*. Three parasite genotypes (0, I, II) occur, with varying degrees of virulence in its host, making it a good model for examining the role of motility in virulence. We compare *C. shasta* cell motility between genotypes and describe how the cellular protrusions interact with the host. We support these observations with motility gene expression analyses. *C. shasta* stages can move by single or combined used of filopodia, lamellipodia and blebs, with different behaviors such as static adhesion, crawling or blebbing, some previously unobserved in myxozoans. *C. shasta* stages showed high flexibility of switching between different morphotypes, suggesting a high capacity to adapt to their microenvironment. Exposure to fibronectin showed that *C. shasta* stages have extraordinary adhesive affinities to glycoprotein components of the extracellular matrix (ECM). When comparing *C. shasta* genotypes 0 (low virulence, no mortality) and IIR (high virulence, high mortality) infections in rainbow trout, major differences were observed with regard to their migration to the target organ, gene expression patterns and proliferation rate in the host. IIR is characterized by rapid multiplication and fast amoeboid bleb-based migration to the gut, where adhesion (mediated by integrin-β and talin), ECM disruption and virulent systemic dispersion of the parasite causes massive pathology. Genotype 0 is characterized by low proliferation rates, slow directional and early adhesive migration and localized, non-destructive development in the gut. We conclude that parasite adhesion drives virulence in *C. shasta* and that effectors, such as integrins, reveal themselves as attractive therapeutic targets in a group of parasites for which no effective treatments are known.

## 1. Introduction

The capacity of movement is fundamental for cells, and most of them rely on a functionally conserved actomyosin cytoskeleton system that allows spatial displacement. The ability to switch plastically between different motility modes and cell protrusions depending on the environment optimizes cell migration. Single cell motility depends on the physical properties of the extracellular matrix (ECM), extracellular proteolysis and signaling factors. Two main modes of migration, i.e., mesenchymal vs. amoeboid can be distinguished by their leading edge structure, cell shape, and the degree of cell adhesion to the ECM. Mesenchymal migration is characterized by polarized and elongated cells that display actin-rich cell protrusions (lamellipodia/sheet-like protrusions and lobopodia), high integrin-mediated adhesion, and proteolysis of the ECM. Amoeboid migration is distinguished by round cells with low or no adhesion that can deform to move cross the ECM without proteolysis, using blebs, pseudopodia and filopodia [1,2,3].

Motility of parasites is essential for their invasion, propagation, immune evasion and disease pathogenesis. Virulence of parasites is strongly linked to their motility and migration strategies, with observed differences in virulence between genotypes and strains with different migration capacities [4,5]. Thus, therapeutic approaches in parasite research target cell motility factors to impair invasion and spread [6,7] and parasite adherence [8] or immune evasion [9].

Myxozoans are microscopic parasitic cnidarians with complex life cycles involving a definitive invertebrate host, usually annelids, and an intermediate vertebrate host, usually teleost fish. As these obligate spore-forming parasites cannot presently be grown in culture, functional studies are limited. Developmental stages of myxozoans are motile during invasion, migration and proliferation [10]. With the exception of the worm-like malacosporeans, in which motility is facilitated by muscle blocks [11], motility in other, more reduced myxozoans depends on the actin cytoskeleton of the primary cell. Movement of *Ceratomyxa puntazzi* [12] and potentially other ceratomyxids [13,14,15,16] occurs through a variety of means such as exploratory filopodia and a leading edge inducing amoeboid crawling. Movement in *Sphaerospora molnari* [17] is promoted by rapidly formed and reabsorbed membrane folds that cause a tumbling, non-directional motility, while *Ceratomyxa vermiformis* uses a nematode-like, undulating movement of the primary cell, without cell protrusions [18]. The study of myxozoan motility deepens our understanding of host-parasite interactions and could aid the development of chemotherapeutic strategies specifically targeting motility factors.

Our model of study is the myxozoan *Ceratonova shasta* (syn. *Ceratomyxa shasta*), which causes severe enteronecrosis in salmonids of cultural and economic value in the Pacific Northwest of North America. The waterborne actinospore invades the fish host through the gills and migrates through the blood to the target organ, the intestine [19]. Once there, it multiplies and invades all layers of the intestine, producing a set of proliferating and spore-forming stages. Myxospores released from the fish host in turn infect the invertebrate polychaete host [20]. The uniqueness of our myxozoan model lies in the existence of *C. shasta* host-associated genotypes (0, I, II) that differ in virulence [21] and Alama-Bermejo et al., *in preparation*. Genotype 0 is a non-lethal, low virulent genotype in rainbow and steelhead trout (*Oncorhynchus mykiss*). Infection by this genotype is usually undetected, as clinical disease does not typically develop, and is only noticeable by the presence of mature myxospores late in the infection [21,22]. In contrast, genotype I and II infections range from moderate to highly virulent depending on host species and strain, parasite dose and environmental conditions. Genotype I infects Chinook salmon (*Oncorhynchus tshawytscha*) and genotype II represents a mix of two types with different host association: IIC for coho salmon (*Oncorhynchus kisutch*) and IIR for rainbow trout (*O. mykiss*) [23,24], and genetically distinct based on our recent transcriptome-wide polymorphisms analysis (Alama-Bermejo et al., *in preparation*).

Salmonid strains that have co-evolved with the parasite generally have a low susceptibility to disease, but strains of the same species from non-endemic rivers are highly susceptible, and provide good models for studying parasite virulence. In susceptible rainbow trout infected with genotype IIR, infections are highly virulent and become systemic, inducing the production of ascites in the peritoneal cavity with consequent massive swelling of the abdomen. This inflammatory fluid is extremely rich in different *C. shasta* stages, can be easily collected and is used routinely to infect naïve fish by intraperitoneal injection as well as to harvest parasites for study [25].

Herein, we describe the three-dimensional morphology, ultrastructure and locomotion of *C. shasta*, and characterize the structural components allowing motility and different types of cell protrusions in the virulent genotypes I and IIR, including novel observations on the behavior of these myxozoan stages on an artificial adhesive surface environment. We hypothesized that virulent genotypes are more active and show a higher level of interaction with its host, e.g., by increased cell protrusion and adhesion, than the low virulent genotype. We compared the course of the infection and determined parasite quantities in gills, blood and intestine of rainbow trout infected with the most virulent genotype (IIR) and the low virulent genotype (0) and then quantified the relative expression of 8 selected genes composing the actomyosin machinery, adhesion complexes and mesenchymal vs. amoeboid motility modes. We demonstrate for the first time the importance of parasite motility and adhesion for the virulence in myxozoans.

## 2. Materials and Methods

### 2.1. Collection of *C. shasta* Genotypes IIR and I for Motility Studies

Between 2013 and 2015, developmental stages of *C. shasta* genotype IIR were collected from ascites, bile and intestine from heavily infected rainbow trout (Roaring River Hatchery strain; *n* = 30; 8.3–31 cm total length) held at the Aquatic Animal Health Laboratory at Oregon State University (AAHL, OSU). Additionally, caeca, liver and testes were collected from three rainbow trout showing gross systemic infection. In June 2015, extremely rare samples of ascites stages of genotype I were collected from five Chinook salmon (Iron Gate Hatchery strain; 7–9 cm total length) after field exposure in the Klamath River (Beaver Creek site), California, USA (these fish do not typically develop ascites during infection). All fish were euthanized by an overdose of buffered MS-222 (tricaine methanesulfonate; Argent Chemical Laboratories, Redmond, WA, USA). Tissues and fluids were collected and processed for different microscopies and molecular analyses.

#### 2.1.1. Light Microscopy and Time Lapse Series

Measurements of stages and cellular processes of I and IIR *C. shasta* were taken as described in [12]. Motile/protruding stages were quantified from 10 µL aliquots of ascites using a counting chamber. Time-lapse images/movies were generated using a Leica DMR microscope (Leica, Wetzlar, Germany) with a Spot RT3 camera, Spot software 5.0 (Spot software, Amsterdam, Netherlands) and VideoPad Video Editor (NCH Software, Canberra, Australia). Recordings ranged from 4 to 18 min, with images captured every 3, 7 or 10 sec.

#### 2.1.2. Confocal Laser Scanning Microscopy

*C. shasta* stages from ascites and intestinal washes were fixed in 4% formaldehyde in 0.1 M phosphate buffered saline (PBS, pH 7.4), for 15 h. Fixed cells were centrifuged (3× at 800 g, 5 min), supernatant was discarded and cells were suspended in PBS. Parasites in PBS were left to settle onto 0.1% poly-D-lysine coated slides for 30 min. Filamentous actin (F-actin) was stained with Alexa Fluor^®^ 488 Phalloidin (495/518 nm, 200 units/mL in methanol, 30 min; Molecular Probes, Eugene, OR, USA). Preparations were mounted with nuclear counterstain Vectashield^®^ Mounting Medium with DAPI (4,6-diamidino-2-phenylindole, dilactate; 1.5 µg/mL, 360/460 nm; Vector Laboratories Inc., Burlingame, CA, USA) and sealed with transparent nail varnish to prevent evaporation of the medium. All CLSM samples were examined using a Zeiss LSM 510 Meta Confocal Microscope System (Carl Zeiss AG, Oberkochen, Germany) at Center for Genome Research and Biocomputing (CGRB, OSU). Projections and Z-stacks were built using Zeiss LSM Image Browser and ImageJ (NIH, Bethesda, MD, USA).

#### 2.1.3. Electron Microscopy (SEM & TEM)

For scanning electron microscopy (SEM), IIR intestinal stages were washed off fish tissue with PBS, collected and fixed with 2.5% glutaraldehyde in PBS. Ascites (I and IIR) and bile (IIR) stages were directly fixed by adding concentrated fixative to the fluid they were in to reach 2.5%. On the day of processing, the parasites were washed in PBS and centrifuged (800 g, 5 min), and prepared as described in [12]. Imaging was performed with a JEOL JSM-7401F (JEOL Ltd., Tokyo, Japan) at the Institute of Parasitology, Czech Academy of Sciences (PARU, CAS) and a FEI QUANTA 600F environmental SEM (FEI, Hillsboro, OR, USA) at OSU Microscopy service. For transmission electron microscopy (TEM), infected intestine, caeca, liver and testes samples were fixed in 2.5% glutaraldehyde in 0.1 M PBS for several days. The tissues were then washed for 1 hr in PBS, post-fixed in 1% osmium tetroxide in PBS for 3 hr and dehydrated in an acetone series, before embedding in Epon resin (Polybed 812, Polysciences Inc., Warrington, PA, USA). Ultrathin sections were cut with diamond knives, stained with 5% uranyl acetate and lead citrate. Stained sections were examined using a JEOL 1010 TEM at PARU, CAS.

### 2.2. Surface Adhesion Experiment of Genotype IIR: 2D Environment

10 µL aliquots of live stages in ascites collected from three different rainbow trout were left to settle onto ethanol-washed 10 µL/mL fibronectin (ThermoFisher Scientific Inc., Waltham, MA, USA) coated slides. Fibronectin is a cell adhesion glycoprotein present in the animal ECM. Control stages were left to settle on uncoated microscopic slides. Both groups were recorded immediately after settling and again after 20 min using a Canon Eos Rebel T1i camera on a Zeiss 47 30 28 light microscope at AAHL, OSU. Videos were analyzed by eye and in vivo behavior of the stages was classified as stages showing mostly (1) filopodia-lamellipodia or (2) blebs. For SEM analysis, 200 µL aliquots of ascites containing live stages were left to settle onto fibronectin coated coverslips for 20 min and fixed in situ with 2.5% glutaraldehyde in PBS. An aliquot of ascites fluid was fixed as a control group. Both fixed stages on fibronectin surface and control fixed ascites stages were processed and visualized as specified above for SEM analysis.

### 2.3. *C. shasta* Genotype 0 and IIR Infections for Transcriptomic Analysis

In May 2016, SPF rainbow trout from Roaring River Hatchery (Scio, OR, Oregon Department of Fish and Wildlife, USA) (length 5.5–7.5 cm; weight 1.6–3.8 gr) were exposed in two different locations within the Klamath county: Keno Eddy (*n* = 60) and Williamson River (*n* = 64). Previous monitoring studies allowed selecting these locations as the most probable source of genotype 0 and genotype IIR infections respectively [22]. Fish were held in mesh cages for 72 h. After exposure, fish were prophylactically treated for the bacterial pathogen *Flavobacterium columnare* during transportation to the AAHL (OSU) and for external parasites within 1-week post exposure [26]. Both groups of fish were held in 100 L tanks in heated 18 °C well water. Fish were monitored daily and mortality was reported as cumulative mortality. Fish were fed regularly with a fasting period of 48 hr before sampling. Five fish per group were sampled at 1, 7, 15, 22 and 29 days post exposure (dpe). An additional time point was collected for type 0 infection 60 dpe. 4–16 µL of blood, all gill arches from one side and the anterior portion of the intestine was frozen at −20 °C for DNA analyses. The other side gills arches and the posterior portion of the intestine was stored in RNA later (Ambion, Austin, TX, USA) and kept at −20 °C. A wet mount of a small portion of distal intestine was examined using bright-field microscopy, and the type of parasite stages present (developmental stages and/or spores) was reported. Naïve fish (*n* = 5) from the same stock were sampled as negative uninfected controls.

To characterize parasite exposure, river water samples were taken at the beginning and at the end of the 72-h exposure for spore dose and genotyping. Three replicates of 1 L water samples were filtered and spore numbers were quantified using a *C. shasta* SSU rDNA-based absolute quantification qPCR assay [27,28] and genotype was confirmed using the ITS-1 rDNA region [29].

### 2.4. *C. shasta* Quantification

In order to compare quantities of parasite in genotypes 0 and IIR infections, an SSU rDNA qPCR assay was performed. First, the DNA content of blood, gills and intestine samples was quantified using the Quant-iT™ dsDNA Assay Kit (Invitrogen, Carlsbad, CA, USA) and a Biotek Synergy HT microplate reader (Biotek, Winooski, VT, USA). Parasite quantities were estimated in 19 ng of DNA from blood (*n* = 15 fish per genotype, 1, 15, 29 dpe), 134 ng from gills (*n* = 15 fish per genotype, 1, 7, 29 dpe) and 50 ng from intestine per reaction (*n* = 25 fish per genotype from all sampling points; *n* = 5 fish per genotype 0 at 60 dpe). These DNA quantities were chosen after quantification of all samples, and taking a value such as all samples could be quantified. *C. shasta* SSU rDNA-based absolute quantification qPCR assay for water samples (Taqman-probe assay) [27,28] was used with modified standards and no IPC test, as no inhibition was observed. A four point 10-fold dilution standard curve of a purified PCR product of a IIR ascites DNA sample was used to calculate SSU rDNA copy numbers of the parasite in the samples analyzed. All samples were run in triplicate, with a positive *C. shasta* sample as an interplate calibrator and a no template control.

### 2.5. Motility Gene Mining from Reference Transcriptome

Motility genes were mined from *C. shasta* reference transcriptome (Alama-Bermejo et al. *in preparation*; NCBI SRA Acc. number SRR6782113). Gene annotations were confirmed by BLASTX searches against three databases: UniProt, Cell Migration Knowledge Database (http://www.cellmigration.org/index.shtml), and CDD (NCBI). Based on cell motility literature, the following genes were selected because their involvement in (1) actomyosin machinery: β-actin (a non-muscle cytoskeletal actin), coactosin, coronin (two actin binding proteins) and myosin-10 (a non-muscle myosin II); (2) cell adhesion: integrin-β and talin; and (3) mesenchymal vs. amoeboid motility regulation: Rac1 (Ras-related C3 botulinum toxin substrate 1) and RhoA (Ras homolog gene family, member A gene). Primers were designed using NCBI/Primer-BLAST [30] (Table S1, Suppl. Fasta file) and their specificity and parasite origin were confirmed using PCR on DNA and cDNA samples of fish infected with genotype IIR and 0, and negative control samples of non-infected rainbow trout. PCR products were purified and sequenced as described above.

As reference genes, a pool of eight *C. shasta* genes were tested by PCR and qPCR: SSU & LSU ribosomal gene regions; EF2 -elongation factor 2-, GAPDH -Glyceraldehyde 3-phosphate dehydrogenase-, NADH -dehydrogenase [ubiquinone] iron-sulfur protein 2-, HPRT -Hypoxanthineguanine phosphoribosyltransferase-, Ornithine aminotransferase and DNA-directed RNA polymerase II (Table S1, Suppl. Fasta file). GAPDH, NADH and HPRT were selected due to their consistently low coefficient of variation (0.8–1.1%). Primer efficiencies were obtained using a set of 2-fold serial dilutions between 5 ng/µL and 0.625 ng/µL and run using qPCR assay described below. The efficiencies were calculated based on the slope of the standard curve in the StepOne^TM^ software, with restriction to ±10% variation (Table S1).

### 2.6. Motility Genes Expression Assays

Intestine samples in RNA later were extracted using a column-based RNA extraction method, High Pure RNA tissue kit (Roche, Basel, Switzerland), including an on-column DNAse step. RNA was quantified using NanoDrop at the CGRB (OSU). Extracted RNA samples were stored at −80 °C. Detection of genomic DNA contamination and quality of RNA was determined by running 100–200 ng of RNA in a 1% agarose gel and using a minus reverse transcriptase control in a subset of samples. 500 ng of RNA was used to synthesize cDNA using the Transcriptor High Fidelity cDNA Synthesis Kit (Roche) and anchored-oligo (dT)_18_ primers. Newly synthesized cDNA was stored at −20 °C.

Five fish intestines per genotype and per sampling time point (7, 15, 22 & 29 dpe) were analyzed for the selected set of motility genes. Three non-infected rainbow trout were analyzed as negative controls. The qPCR reaction mix consisted of 5 µL TaqMan^®^ Universal PCR Master Mix (Applied Biosystems, Foster City, CA, USA), 1:100 dilution SYTO^®^ 9 Green Fluorescent Nucleic Acid Stain (Molecular Probes) in 1× TAE buffer, 10 µM of each primer, 25 ng/µL BSA, 5 ng of total cDNA and up to 10 µL of PCR grade water. 96 well plates were run and read using a StepOnePlus qPCR machine (Applied Biosystems) with the following cycling conditions: polymerase activation at 50 °C for 2 min, denaturation at 95 °C for 10 min, 44 cycles of denaturation at 95 °C for 15 s and annealing at 60 °C for 1 min, and a final melt curve stage of 95 °C for 15 s, 64 °C for 1 min and 88 °C for 15 s, in order to detect any unspecific PCR product. All qPCR reactions were simplex and run in triplicate. A positive *C. shasta* sample was run in all plates with NADH gene assay as an interplate calibrator, to compensate for the variation in qPCR runs (Cq +/-0.5). All plates were run with no template control and ROX was used as passive reference.

A Cq mean was calculated for each sample. Relative gene expression is shown as fold change using 2^−ΔΔCq^ method [31], assuming the low virulent genotype 0 is the calibrator and the highly virulent genotype IIR is the treated group (ΔΔCq = [(C_q_ gene of interest − C_q_ average three reference genes) genotype IIR − (C_q_ gene of interest − C_q_ average three reference genes) genotype 0]). Additionally, intragenotype temporal changes were calculated as relative change (2^−ΔCq^) to the reference genes. Differences between mean fold changes and relative change were tested for statistical significance using Tukey’s method for multiple comparisons after one-way ANOVA or t-test for normally distributed data or Kruskal-Wallis with Dunn’s multiple comparison in non-normally distributed data (Tables S3 and S4). All statistical analysis and graphs were done using SigmaPlot 13.0 (Systat Software Inc., Chicago, IL, USA).

## 3. Results

### 3.1. Motility Modes in *C. shasta*—Blebbing, Adhesion and Crawling

*C. shasta* showed asynchronous development in its fish hosts. Early and late sporogonic stages as well as mature myxospores were observed in intestine, ascites, bile, liver, caeca and testes. Proliferating stages of *C. shasta* measured 9.9–92.8 µm in length (*n* = 88) and showed high morphological plasticity, with round or ellipsoidal shape, occasionally pyriform, and a variety of cell protrusions and active motility. Three main types of cell protrusions were observed on the outer, or primary, cell of IIR *C. shasta* stages: blebs, filopodia and lamellipodia. These cell protrusions were associated with different behaviors: (a) blebbing-driven movement with little to moderate displacement and low adhesion; and (b) lamellipodia-driven movement with high adhesion. The latter type of movement may be characterized either by absence of/little displacement, and abundant filopodia or by directional (crawling) movement and few filopodia in uropod. Stages could switch between protrusions and migration modes.

### 3.2. Myxozoan Blebbing Promotes Fast Amoeboid Motility and Spore Release

**Figure 1:**
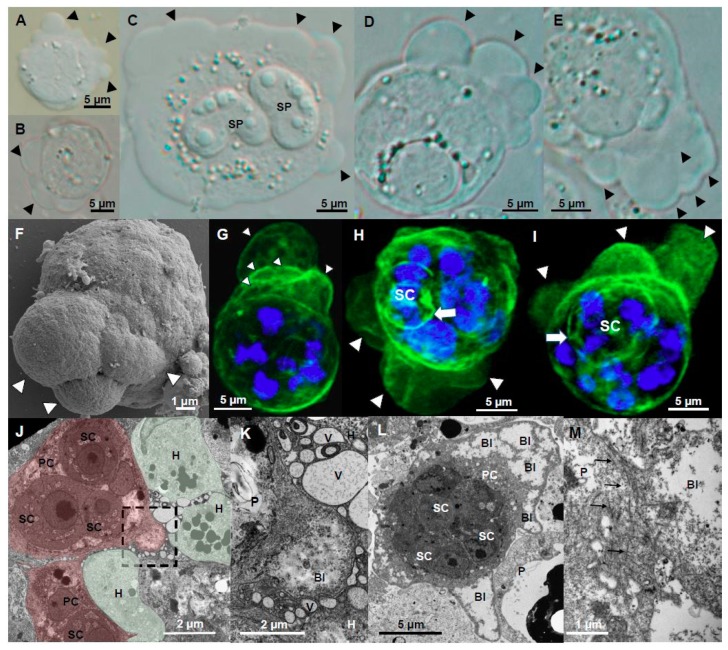
Bleb protrusions on *Ceratonova shasta* genotype IIR stages in rainbow trout. (**A**,**B**) Small presporogonic stages showing active blebbing. (**C**–**E**) Sporogonic stages showing active polarized blebbing, in (**C**) stage contains two myxospores. (**D**,**E**) Overlapping blebs, concentrated at one side of the stage. (**F**) External appearance of 3 overlapping blebs. (**G**–**I**) F-actin distribution on blebbing stages. (**G**) Overlapping blebs. (**H**,**I**) Blebs of different sizes, probably showing blebs at different stages of their life cycle. Notice secondary cells, probably containing tertiary cells, that showed F-actin labelled membranes. (**J**) Parasite stage (in red) containing three secondary cells visible in the caeca surrounded by host cells (in green) and other parasite stages, showing a hemispherical bleb (square) being protruded between host cells. Notice the lack of cytoplasmic content in the bleb. (**K**) Detail of the bleb on J, notice the bleb is surrounded by host vesicles. (**L**) Parasite stage containing three secondary cells in the intestine showing profuse blebbing; notice the lack of cytoplasmic content of the blebs. (**M**) Base of a bleb in a liver parasite stage, with abundant actin filaments. Head arrows: blebs, White arrow, F-actin in secondary cell. Black arrows, actin filaments. **A**, **F**, Bile; **B**–**E**, **G**–**I**, Ascites; **J**,**K** Caeca; **L**, Intestine; **M**, liver. **A**–**E** Light microscopy, **F** SEM, **G**–**I** CLSM (Green-F-actin, phalloidin; blue-nucleic acids, DAPI), **J**–**M** TEM. SP-spore; P-parasite; H-host; PC-primary cell; SC-secondary cell; Bl-bleb; V-vesicle.

**Figure 2:**
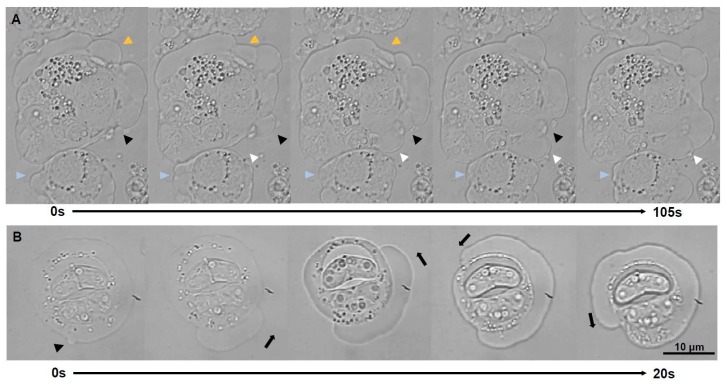
Light microscopy time-lapse series of blebbing stages of *Ceratonova shasta* genotype IIR from ascites of rainbow trout. (**A**) Polarized blebbing (total time elapsed 105 s, images every 21 s), head arrows point to surface positions of the stages where blebs are expanding and rectracting, with physical overlap of several blebs simultaneously. (**B**) Non-polarized blebbing or circus movement (total time elapsed 20 s, images every 5 s): a bleb expansion initiated and propagated along the circumference of the sporogonic stage (with 2 mature spores), fully circumnavigating the parasite.

This is the first time blebbing is reported for myxozoans, although this type of amoeboid migration has a starring role in other cells e.g., amoeba, embryonic cells and tumor cells [32]. Blebs (Figure 1A–I) were hemispherical hyaline cell protrusions of highly variable size (2.4–52.4 µm width; 2.3–45.2 µm height; *n* = 62) and short life span (mean 20 s, range 6–36 s). Blebbing stages were active but showed little physical displacement (Movie 1). Blebs consisted of semicircular membrane structures with little cytoplasmic content (Figure 1J–L). Scattered F-actin was concentrated on the primary cell membrane, at the base and/or on the surface of blebs (Figure 1G–I), probably indicating blebs in different moments of their life cycle, i.e., initiation, growth and retraction [33]. Unlike filopodia and lamellipodia, blebs are produced by hydrostatic pressure created by the actomyosin cortex [32,34]. Blebs protruded between host cells in intact and degraded ECM (Figure 1J,L), probably aiding parasite migration through the gaps of the matrix using blebs to push and squeeze their way [35]. In some cases, host cells surrounding the parasite showed abundant intracellular vesicles (Figure 1J,K). Actin microfilaments were visible in ultrathin sections at the base of blebs (Figure 1M). Blebs expanded rapidly then retracted more slowly until they completely disappeared. Blebs were observed to occur randomly in location and time on the membrane. Blebbing is considered an alternative to lamellipodial amoeboid migration but in contrast to filopodia and lamellipodia, with little adhesion, displacement or directionality [33]. Two blebbing patterns were observed to occur: polarized-directional and non-polarized. Polarized blebbing stages predominantly protruded blebs on one pole of the parasite (Figure 2A). Blebs would continuously expand and retract, often with physical overlap of several blebs simultaneously at the same location (Figure 1D–I, Movie 1). Sometimes the blebbing pole was found to shift to another side of the parasite, or blebbing completely ceased. Non-polarized blebbing, or circus movement, was less common in *C. shasta* and it consisted of a massive cell membrane detachment that was initiated as a regular hemispherical bleb, which propagated rapidly and progressively around the surface of the stage, returning to the initiation point (Figure 2B, Movie 2). Circus movement has been reported in embryonic blastomeres of lower vertebrates [36], probably involved in embryonic cell migration [37]. Full circumnavigation lasted 19–27 s and the circular surface expansion could repeat several times (up to 3 observed). While polarized blebbing stages were observed to create some degree of physical displacement, no displacement was observed for the stages with circus blebs and its functionality for *C. shasta* stages is unclear.

Blebbing was long considered a hallmark of cell apoptosis [32] and it may play an apoptotic role in *C. shasta* sporogonic stages, at a later stage of development. In some mature plasmodia containing myxospores, blebbing stages expelled single mature spores (Movie 3). In others, active blebbing happened before bursting and releasing the spores from the primary cell (Movie 4).

### 3.3. Filopodia and Lamellipodia Promote Myxozoan Adhesion While Adhesive Surfaces Promote the Formation of These Cell Protrusions

**Figure 3:**
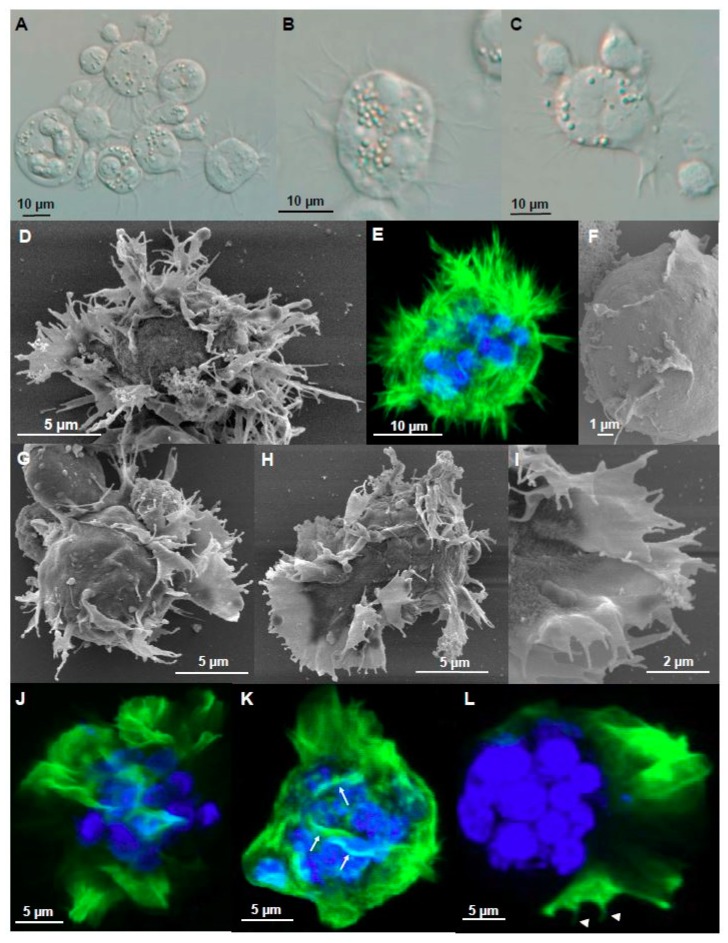
Filopodia and lamellipodia of adhesive stages of *Ceratonova shasta* genotype IIR in rainbow trout. (**A**) Presporogonic and sporogonic developmental stages showing abundant static filopodia. (**B**) Radially distributed filopodia on a stage. (**C**) Sheet-like lamellipodia with filopodia on the lamellipodia border and also all over the body of the stage. (**D**) 3D radiating pattern of filopodia. (**E**) F-actin distribution on a 3D radiating pattern of filopodia. (**F**) Detail of the parasite surface with small crests. (**G**,**H**) Parasite stages with sheet-like lamellipodia and small filopodia projecting in the external margin. Notice the sheet-like lamellipodia located on one side, with opposing single and ramified filopodia over the rest of the parasite body. (**I**) Detail external margin of lamellipodia with small filopodia on the external border. (**J**–**L**) F-actin distribution on stages with lamellipodia. (**K**) Stage showing F-actin rich small crests (arrows) on the surface. (**L**) Small F-actin rich filopodia (head arrows) on the external margin of the lamellipodia. All stages were collected from ascites. **A**–**C** Light microscopy, **D**, **F**–**I** SEM, **E**, **J**–**L** CLSM (Green-F-actin, phalloidin; blue-nucleic acids, DAPI).

**Figure 4:**
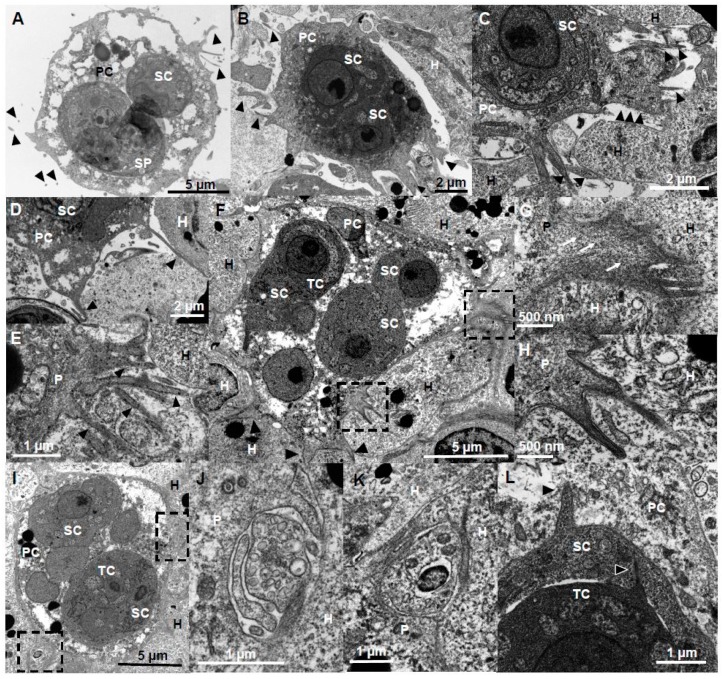
Ultrastructure (TEM) of cell protrusions of adhesive stages of *Ceratonova shasta* genotype IIR in rainbow trout. (**A**) Sporogonic stage, containing a forming spore and a secondary cell, showing small thin filopodia protruding from the primary cell. (**B**) Parasite stage containing two secondary cells with abundant pseudopodia being projected into the ECM. (**C**) Plasmodia showing a large sheet-like lamellipodia and filopodia protruding into a degraded ECM. (**D**) Parasite stage protruding two long and thin filopodia between host cells. (**E**) Group of filopodia being projected from a parasite. (**F**) Large parasite stage containing two primary nuclei, three secondary cells, one of them with a tertiary cell, with filopodia and lamellipodia deeply embedded (anchored) in the surrounding host cells (boxes). (**G**,**H**) Detail of the the filopodia-lamellipodia in F, showing a mesh of actin filaments supporting the protrusion. (**I**) Parasite stage presumably feeding by endocytosis, using cell protrusions to capture small portions of host cells. (**J**,**K**) Endocytosis processes in (**I**), small parasite protrusions engulfing host cell fragments. (**L**) Detail of a parasite stage showing small filopodia being protruded by the secondary cell into the primary cell, and by the tertiary cell into the secondary cell. **A**, **B**, **D**, Intestine; **C**, **E**, **I**–**L**, caeca; **F**–**H** testes. Head arrows, filopodia and lamellipodia; black arrows, actin filaments; SP, spore, PC, primary cell, SC, secondary cell, TC, tertiary cell, H, host cell, P, parasite.

Filopodia and lamellipodia have important roles in cell migration and adhesion, including substrate tethering and environment probing [38]. They are typical for cells migrating in a mesenchymal mode, i.e., irregularly shaped cells with strong adhesion by integrin and degradation of the ECM [39]. These cell protrusions have previously been reported for other myxozoans e.g., at the anterior pole of freely swimming bile stages of *C. puntazzi* [12]. Filopodia and lamellipodia played a major role in *C. shasta* adhesion. Adhesive stages were motionless, with filopodia and lamellipodia as abundant cell protrusions that increase surface contact of otherwise static cells to the host ECM (Figure 3 and Figure 4). Filopodia were F-actin rich, 1.3–24.1 µm in length and 0.3–1.8 µm (*n* = 68) in thickness and were often ramified and distributed in a 3D radiating pattern or star-like arrangement (Figure 3A,B,D and Figure 4A). Lamellipodia (Figure 3C,G–I) were flat F-actin rich sheet-like protrusions (Figure 3J–L), observed frequently with small filopodia projecting from the margin of the cell (Figure 3G–I,L). Usually, these lamellipodia were located on one side, with single and ramified filopodia present on the remainder of the parasite body (Figure 3C,G,H). Some stages showed small F-actin-rich crests on the surface (Figure 3F,K). Intraepithelial stages in the intestine, caeca and testes showed sheet-like lamellipodia and filopodia that extended between host cells in a degraded ECM (Figure 4B,C) probably probing the environment, guiding cell migration by connecting the parasite cytoskeleton with the host ECM [40] while also feeding on host proteins. Some of the stages had thin and long single filopodia (Figure 4D) whereas others had groups of filopodia extending between host cells (Figure 4E). In testes, these cell protrusions deeply anchored the parasite into host cells, resulting in parasites completely embedded in otherwise intact tissue (Figure 4F–K). These protrusions were supported by a mesh of actin filaments (Figure 4G,H). Cell protrusions were mainly exhibited by the primary cell, however, small filopodia were observed on secondary and tertiary cells protruding into the primary and secondary cells respectively (Figure 4L).

**Figure 5:**
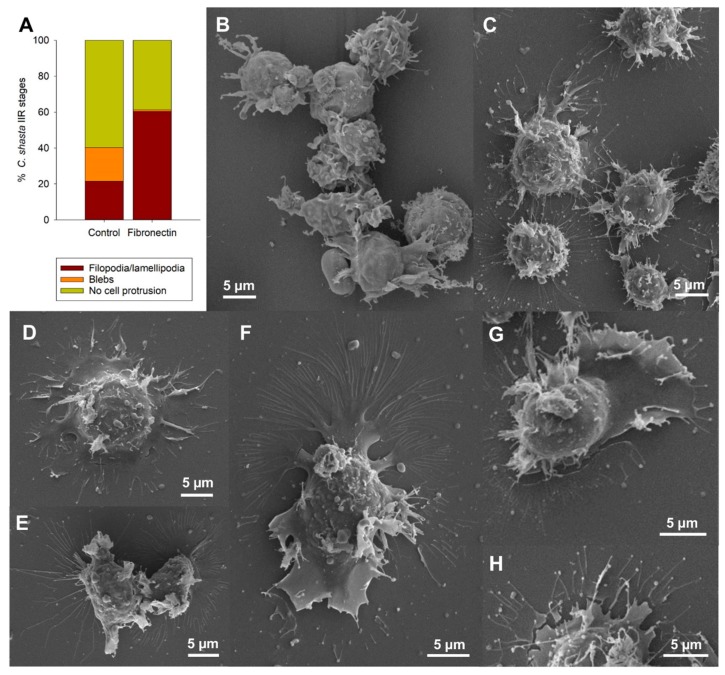
Adhesion experiment with *Ceratonova shasta* genotype IIR stages collected from ascites of rainbow trout. (**A**) Percentage of stages exhibiting different cell protrusions on control non-coated slides and on fibronectin coated slides. (**B**) Control stages showing a 3D distribution of filopodia and lamellipodia. (**C**) Stages on fibronectin showing a strong adhesion for the surface with a 2D distribution. (**D**) Stages on fibronectin with radially distributed lamellipodia with small filopodia on the external margin. (**E**) Two stages partially attached, one of them with long and thin filopodia. (**F**,**G**) Polarized stages on fibronectin with sheet-like lamellipodia on one pole and long and ramified filopodia on the other pole. (**H**) Detail of slightly thickened filopodia tips of an adhesive stage. **B**–**H**, SEM.

Parasite stages exposed to an adhesive 2D substrate (i.e., ECM binding protein fibronectin) showed extraordinary high attraction and binding activity to the adhesive surface. An increased occurrence of filopodia and lamellipodia was observed in 60.4% (136/225) of the parasites on fibronectin, whereas only 21.6% (53/245) showed these protrusions on uncoated slides (Figure 5A). Formation of blebs was discontinued in parasites exposed to an adhesive surface, with only 0.8% (2/225) of them blebbing, in contrast to 18.6% (46/245) in the control group, supporting their role in an amoeboid motility mode that requires little adhesion. Using SEM, the distribution pattern of filopodia-lamellipodia changed from a 3D distribution in the control stages (Figure 5B) to a 2D distribution in stages on fibronectin (Figure 5C–H), with a strong affinity for the coated surface. The protrusions of stages on fibronectin extended radially 1.9 to 14.9 µm from the body surface (similar to the filopodia length of non-treated stages). Parasite adhesion was complete (Figure 5D) or partial (Figure 5E). Radially distributed lamellipodia showed further filopodia projected from the external margin (Figure 5D). Polarized stages showed a large sheet-like lamellipodium on one pole and filopodia on the other side (Figure 5F,G). In some cases, stages showed long ramified filopodia extending over large areas (Figure 5F). Some filopodia showed slightly thickened tips (Figure 5H).

### 3.4. Crawling Stages: Fast and Directional Motility

Crawling stages showed active displacement (Movie 5) but were the least frequent form of motility (5.3%; 10/188). When performing this motility, the usually round stages would stretch and acquire a pyriform shape, with an active round anterior edge. The posterior end or uropod was static and dragged along by the anterior end. The uropod possessed several short static filopodia with a root-like appearance. The leading edge position varied, dragging the parasite in different directions. Strong cytoplasmic streaming was observed in the primary cell of the stage, with secondary cells and/or sporoblasts sometimes containing mature spores that were moved or pushed within the primary cell to the anterior pole. The speed of displacement was observed to be between 5.4–19.4 µm/min. We did not determine the F-actin distribution in crawling stages.

### 3.5. Motility Mode Switching Optimizes *C. shasta* Migration in Complex Environments

*C. shasta* IIR stages showed the ability to switch between different cell morphologies, protrusions and motility types. Rapid transitions were observed between blebs, lamellipodia and filopodia, in different combinations. Alternation of blebs and actin-rich protrusions is common in 3D environments and some cells use this ability for a directed, more precise migration [41]. Parasites could switch between blebbing and lamellipodia with filopodia (Movie 6), or change from polarized to circus blebbing (Movie 1). The reversible nature of blebbing and the long-term survival of blebbing cells are signs of blebs involved in cell motility [42]. The reversible nature and long-term survival of blebs observed in *C. shasta* support the non-apoptotic role of this form of motility. In some cases, *C. shasta* stages had both blebs and actin-rich protrusions, static filopodia, simultaneously (Movie 6) or could transition to motionless stages with filopodia, and stop all visible displacement. Plasticity in cell protrusion formation is thought to optimize cell migration in complex environments (e.g., during embryos development, cell chemotaxis) and to promote cancer dissemination [32].

### 3.6. Physical and Morphological Differences in Motility-Related Structures Exist Between *C. shasta* Genotypes

**Figure 6:**
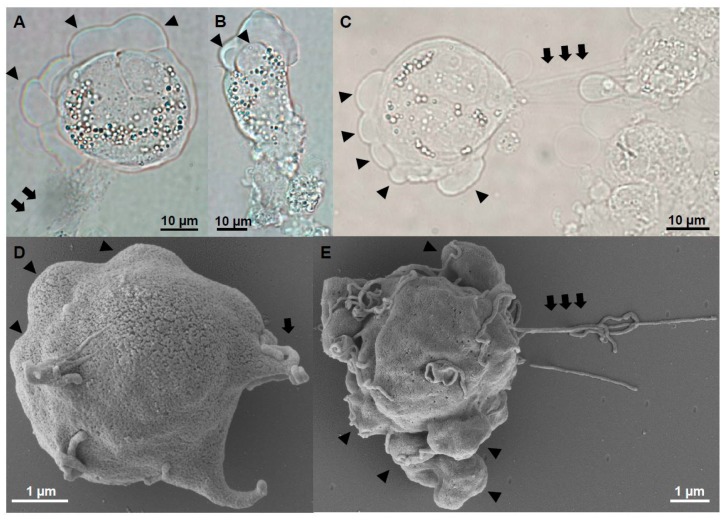
Motile stages of *Ceratonova shasta* genotype I from ascites of Chinook salmon. (**A**–**E**) Stages showed profuse blebbing at the anterior end and a posterior end with extensible filaments, which anchored the stage to other cells. Head arrows: blebs, Arrows: filaments. **A**–**C**, Light microscopy. **D**,**E**, SEM.

Motility of type I *C. shasta* stages from Chinook salmon differed from type IIR stages from rainbow trout. While possessing the same type of cell protrusions, i.e., lamellipodia, filopodia and blebs, type I stages were strongly directional with all protrusions simultaneously produced at the anterior pole. We did not observe type I stages that displayed only blebbing or crawling behavior, nor represented adhesive stages exclusively, as observed for IIR. Type I migrating stages were pyriform to round (Figure 6A–C), with two well-defined ends: (1) a leading edge, with large and profuse blebbing and lamellipodia/filopodia and (2) a posterior end with long and extensible filaments that acted as a root or uropod, anchoring the stage to host cells or other parasites (Movie 7). The posterior filaments were unique to type I and were observed using SEM (Figure 6D,E). Parasites were able to migrate, pushing and moving forward between host cells using this configuration (Movie 7) at a speed of 3.6–5.8 µm/min and hence slower than type IIR crawling stages (see above). With this combination of cell protrusions, these stages showed an exploratory behavior in the ascites, rather than targeted migration (Movie 8). We previously observed that a large genetic divergence of cell migration genes exists between genotypes I and IIR (Alama-Bermejo et al., *in preparation*) and these differences may be reflected in considerable differences in their migration phenotypes.

### 3.7. Low Proliferation and Delayed Spore Production Characterize Low Virulent Genotype Infections; Fast and Massive Proliferation Characterizes Virulent Genotypes

**Figure 7:**
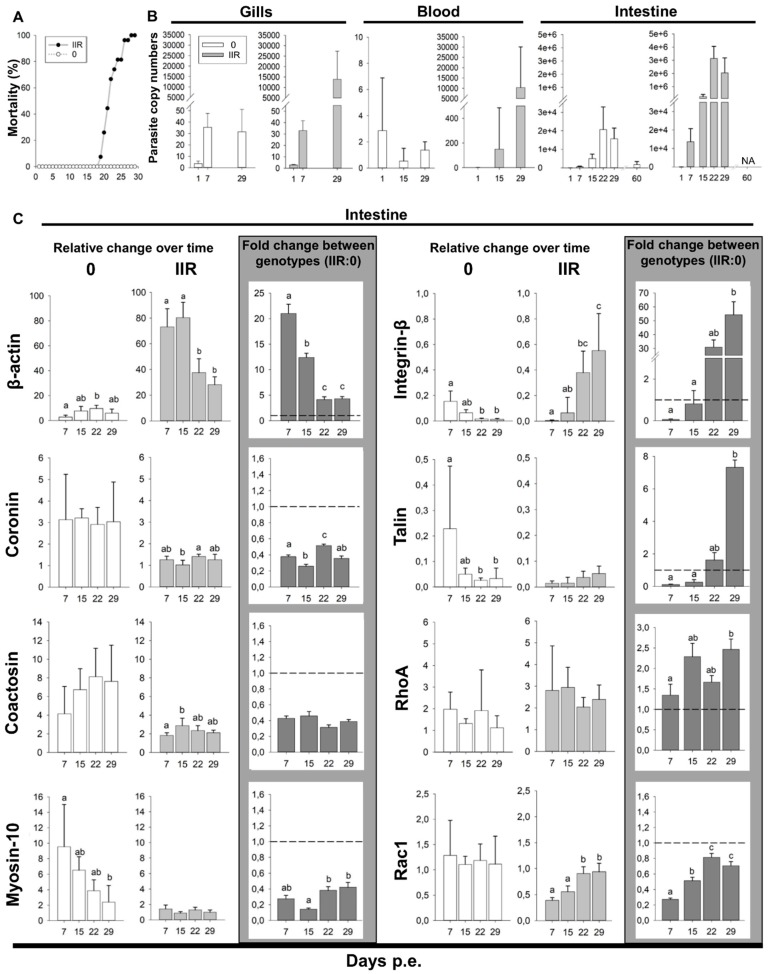
*Ceratonova shasta* genotypes 0 and IIR experimental infection dynamics in rainbow trout and motility genes expression. (**A**) Mortality curve (%) for type 0 and IIR over the course of the infection. (**B**) Parasite SSU rDNA copy numbers (qPCR) in the gills, blood and intestine for genotype 0 and IIR at different days post exposure (dpe). (**C**) Parasite motility genes expression in the intestine: Relative change (2^−ΔCq^) for each genotype over time and fold change (2^−ΔΔCq^) between genotypes (IIR:0).

The disease dynamics were markedly different between genotypes. Low virulent genotype 0 fish showed no clinical signs, no parasite stages were observed microscopically during the first month and there were no mortalities. We detected mature spores in the feces of otherwise healthy fish three months following exposure. Genotype 0 infection was confirmed by genotyping. Mortality in genotype IIR infected fish, first occurred on 19 dpe, peaked on 22 dpe (6 fish) (Figure 7A), and reached 100% on 28 dpe. The first clinical signs in these fish occurred on 15 dpe, with enlarged intestine and whitish liver. Early parasite stages were observed microscopically in 1/5 fish on 15 dpe. On 22 dpe, all fish were heavily infected, with swollen abdomen, ascites, hemorrhagic liver with white nodules, and whitish and enlarged intestine. By 29 dpe, fish became lethargic and emaciated. Internal organs looked similar to 22 dpe infection, except for the kidney, which became enlarged and with white nodules. Microscopically, sporogonic stages and mature spores were observed between 22 and 29 dpe. On 29 dpe, mature spores were predominant in the gut.

Parasite dose was approximately 18 spores/L for genotype 0 at both sampling points, while genotype IIR was undetected at the beginning but measured 16 spores/L at the end of the exposure. Gills were PCR positive through all sampling days for both genotypes. 1, 7 and 29 dpe gill samples were quantified for parasite copy numbers (Figure 7B, Table S2) revealing similar copy numbers on 1 and 7 dpe for both genotypes. On 29 dpe, genotype 0 levels in the gill remained unchanged while genotype IIR increased nearly 400-fold. Detection of the parasite in blood (samples quantified on 1, 15 and 29 dpe) was less than 1 copy for both genotypes on 1 and 15 dpe (Figure 7B). On 29 dpe, genotype 0 remained low in the blood while IIR was detected at a higher copy number, but with high variability (Table S2). Intestine was PCR positive for both genotypes except on 1 dpe. Parasite quantities in the intestine (Figure 7B, Table S2) followed a similar trend for both types: numbers increased over time, peaking on 22 dpe. However, intestinal parasite copy numbers of IIR infected fish was 21 to 152-fold higher than in genotype 0 fish. Type 0 copy numbers decreased 9-fold between 29 and late 60 dpe.

### 3.8. β-actin, Integrin-β, Talin and RhoA Are Upregulated in *C. shasta* Virulent Genotypes

Comparison of motility gene expression in the intestines of rainbow trout infected with virulent genotype IIR relative to low virulent genotype 0 revealed that four genes were upregulated: β-actin, integrin-β, talin and RhoA, with the highest fold changes observed for integrin-β (up to 54-fold change) and β-actin (up to 21-fold change) (Figure 7C, Table S3).

β-actin was the only actomyosin machinery-related gene showing significant upregulation at all time points in IIR infections, ranging between 4- to 21-fold change. Coronin, coactosin and myosin-10 were downregulated throughout the infection. Cell adhesion gene integrin-β showed the highest fold changes in this study, with 31- and 54-fold increases late in the infection (22 and 29 dpe respectively). Talin showed a similar pattern, with a 7-fold change on 29 dpe. RhoA was the only motility regulator gene upregulated, with up to 2-fold change (15 and 29 dpe.). Rac1 was downregulated throughout the infection.

The comparison of gene expression over time between genotypes revealed further differences and opposite trends (Figure 7C, Table S4). β-actin expression was extremely high at the beginning of the infection with virulent type IIR (7 and 15 dpe), while it was highest on 15 and 22 dpe in the low virulent type 0. Myosin-10 decreased over time in 0 but showed no clear trend in IIR. In type 0 infections, the genes involved in cell adhesion, integrin-β and talin, showed a significant change in expression levels over time, with the highest expression on 7 and 15 dpe, followed by a decrease on 22 and 29 dpe. In contrast, a strong increase of integrin-β and a moderate increase of talin expression at these latter timepoints was observed in IIR infections. This indicates a high recruitment of adhesion-related genes for the virulent type IIR, during later stages of infection. Amongst the genes involved in cell motility regulation, RhoA showed no significant differences over time for any genotype and expression of the gene involved in mesenchymal motility regulation, Rac1, increased over time for IIR with significant difference between early and late time points.

## 4. Discussion

**Figure 8:**
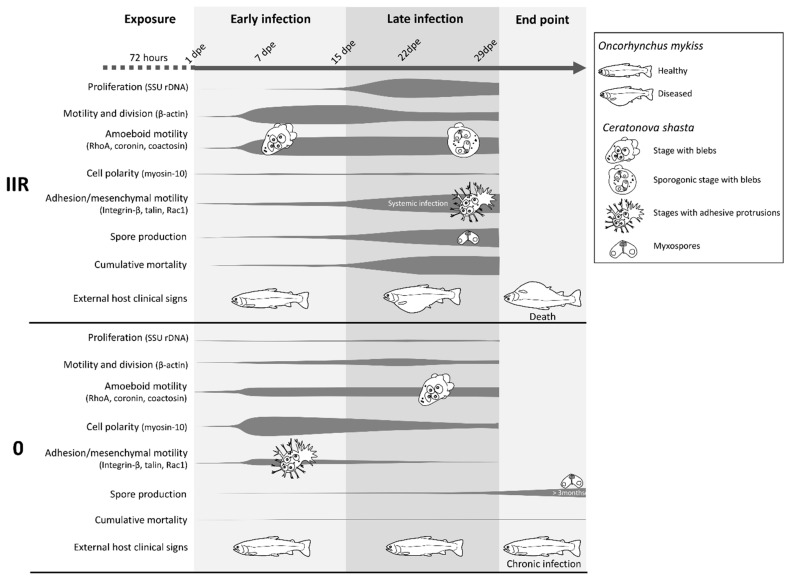
Model summarizing the infection dynamics, parasite motility and proliferation of *Ceratonova shasta* genotypes IIR and 0 in the intestine of rainbow trout, combining results from visual observations, parasite molecular quantification and gene expression, and host clinical signs.

The parasitic cnidarian *C. shasta* develops and proliferates intercellularly in all layers of the intestine [19] resulting in a high level of contact and interaction with the host ECM. The present observations of *C. shasta* motility/protrusion modes and differential motility gene expression in different genotypes provide a first comprehensive characterization of the toolbox enabling migration and demonstrate morphological, cell multiplication and behavioral differences between virulent and avirulent genotypes of these parasites (Figure 8, Table S5).

### 4.1. Fast Proliferation and Rapid Bleb-Based Migration Characterize Virulent *C. shasta* Strain Invasion

We demonstrate that both migration strategies and rates of parasite proliferation differ between virulent and less virulent genotypes of *C. shasta,* as reported for other pathogens [4,43]. While both type IIR and 0 managed to establish and multiply in their fish host, IIR proliferated more rapidly during early infection in blood, gills and intestine. This difference could be an adaptation of virulent genotypes that allow the parasite to complete its development, and hence its transmission, before the host can respond immunologically [44]. The higher and early expression of β-actin in the virulent type suggests more motile and/or dividing stages during early infection, which could contribute to initial fast growth, multiplication and spread of the parasite [45].

In the virulent IIR genotype, rapid blebbing appears to be the chosen migration mode in an intact intestinal ECM. Downregulation of coronin (controller of actin subunits flux) [46] and coactosin (actin polymerization) [47] suggests that this genotype favors non F-actin rich amoeboid migration, such as blebbing motility. Furthermore, RhoA upregulation in IIR suppports non-lamellipodial amoeboid motility as the preferred mode in the virulent genotype, as high levels of RhoA in the cells inhibit lamellipodial-based migration and induce a switch to bleb-based cell migration [48]. As this represents a faster migration mode than mesenchymal migration [39], it may further explain why the parasite is able to reach and spread quickly in the intestine, using blebs to push and squeeze their way through the gaps.

### 4.2. Virulent Genotypes Interact with and Disrupt ECM at Late Stage Infection

Virulent IIR *C. shasta* infections are associated with massive destruction and loss of intestinal epithelium structure, with associated host mortality. The disease outcome is probably caused by a simultaneous effect of different factors affecting the ECM structure: parasite characteristics (proteolysis, feeding, adhesion) and host immune responses (inflammatory response, remodeling of ECM), as reported for other pathogens [49]. *C. shasta* adhesive structures likely play a very important role in shaping the virulence of the parasite at this stage of the infection. We demonstrate that *C. shasta* filopodia and lamellipodia have very strong affinity for glycoprotein components of the ECM, such as fibronectin. Parasite adhesion factors integrin-β and talin are upregulated late in the infection, and appear to be important in inducing changes in the ECM. Changes in adhesive substrates can induce cell haptotaxis, which is mediated by integrins. This cell movement plays important roles in tumor cell dissemination [50] and may be equally important in parasite dissemination. Interestingly, upregulation of these genes in IIR coincides with the change from an intestine localized infection to dispersion into and proliferation in other organs, i.e., liver, kidney, testes.

Active lamellipodia-based migration requires Rac1-mediated actin polymerization [51]. Rac1 was downregulated in genotype IIR, especially during initial infection, suggesting that lamellipodial-based migration is not the preferred motility mode for the virulent genotype. However, this GTPase showed a significant increase over time for IIR which suggests an increased use of lamellipodia and potentially a more proteolytic mesenchymal migration mode for the virulent genotype, during late infection. Together, these findings suggest that pathogenesis of IIR stages is likely related to a high level of interaction with the ECM (upregulated adhesion factors). Modulation and destruction of the ECM by means of adhesion is probably facilitating feeding and proliferation of genotype IIR, potentially promoting its haptotactic motility and consequent dispersion (systemic infection) and is the reason for its differential pathogenic capacity.

### 4.3. Early Direction-Driven Invasion Followed by Low Proliferation and Slow Mesenchymal Migration to Target Site Characterizes Low Virulent *C. shasta*

While the virulent *C. shasta* genotype IIR has been intensively studied due to its effects on rainbow trout health, this is the first attempt to unravel the biology of the less virulent type 0 in a comparative approach. The infection strategy of type 0 is characterized by low proliferation rates, less active stages and a delayed parasite development. This is revealed by low parasite copy numbers, downregulation of β-actin expression and long-term spore release (first spores observed after three months and up to 2 years pe, Atkinson & Bartholomew, personal communication).

Despite the low proliferation rate and less active stages of the low virulent genotype, these stages seem to perform strong directional and adhesive migration in the first stages of infection in the intestine. Upregulation of adhesive factors integrin-β and talin early in the infection suggests that mesenchymal migration may have a relevant role during invasion. Mesenchymally migrating cells acquire an elongated shape with a leading edge [39]. Type 0 stages showed early increase in expression of myosin-10, a gene involved in cell migration direction (front-to-back), suggesting a strong targeted migration during invasion, coordinating protrusion and stabilizing cell polarity [52,53]. In contrast, the frequently undefined polarity (cell protrusions projected in different directions) of IIR stages and the overall downregulation of its myosin-10 expression suggests cell polarity is not of major importance to virulence in *C. shasta*.

### 4.4. Moderate Exploitation of Target Tissues by Less Virulent Genotype

The initial mesenchymal migration strategy is abandoned later in the infection of type 0, with decreased expression of both adhesion factors and myosin-10. After initial invasion of the intestine, type 0 stages appear to proliferate slowly and form spores in the gaps of the ECM, while IIR proliferates rapidly and spreads widely. These differences suggest that type IIR stages are more active in the target organ than type 0, which may be related to the ability to respond to nutrient depletion and outgrowth of their own metabolism. Parasites like *Entamoeba histolytica* Schaudinn, 1903 show increased motility as a response to nutrient depletion and repellence by their glycolysis by-products [54]. In a cycle of cause and effect, increased proliferation requires more resources, forcing stages to migrate to other areas and organs where they can continue to feed and reproduce, thereby increasing pathogenesis and virulence.

Recent findings show that genotype 0 does not elicit an evident host immune response (Taggart-Murphy et al. *in preparation*) which together with phenotype, migration behavior and proliferation rate of this genotype likely indicates a high level of host-parasite mutual adaptation. This strengthens the hypothesis that the relationship between rainbow trout and *C. shasta* genotype IIR is out of balance due to the relatively recent encounter of the parasite and this new naïve host in which the parasite multiplies in an uncontrolled manner.

## 5. Conclusions

This study revealed the great diversity of morphologies, motility types and protrusions of the parasitic cnidarian *C. shasta* in its salmonid hosts. The phenotypic plasticity and the parasites’ ability to switch between motility modes suggests a high capacity for adaptation to a changing microenvironment. Differential morphology and gene expression patterns in *C. shasta* genotypes characterized by different degrees of virulence revealed that parasite adhesion and increased spread represents an important pathogenic mechanism that shapes myxozoan virulence. Virulent genotype IIR is characterized by fast initial proliferation, initial rapid bleb-based migration, followed by increased parasite adhesiveness with massive interaction and disruption of the host intestinal ECM at late stage infection. The less virulent genotype 0 is characterized by low proliferation rates and slow direction-driven mesenchymal migration, without massive exploitation of target tissues. Myxozoan integrins are spotlighted as attractive chemotherapeutic intervention targets, due to their essential role in virulent interactions, as well as their known function in leukocyte homing, inflammation and cancer. Anti-integrin therapies have been successful in gut-related diseases such as inflammatory bowel disease [55]. As a first step to controlling of the enteronecrosis disease in salmonids we need to obtain a better understanding of the reciprocal feedback between *C. shasta* parasite cells, host ECM and the immune system.

## Supplementary Materials

The following are available online at https://www.mdpi.com/2076-2607/7/10/397/s1.

## References

[1] Friedl P., Wolf K. (2010). Plasticity of cell migration: A multiscale tuning model. J. Exp. Med..

[2] Petrie R.J., Yamada K.M. (2012). At the leading edge of three-dimensional cell migration. J. Cell Sci..

[3] Petrie R.J., Yamada K.M. (2016). Multiple Mechanisms of 3D Migration: The Origins of Plasticity. Curr. Opin. Cell Boil..

[4] Barragan A., Sibley L.D. (2002). Transepithelial Migration of *Toxoplasma gondii* is Linked to Parasite Motility and Virulence. J. Exp. Med..

[5] Marie C., Petri W.A. (2014). Regulation of Virulence of *Entamoeba histolytica*. Annu. Rev. Microbiol..

[6] Heaslip A.T., Nishi M., Stein B., Hu K. (2011). The Motility of a Human Parasite, *Toxoplasma gondii*, is Regulated by a Novel Lysine Methyltransferase. PLoS Pathog..

[7] McCammick E.M., McVeigh P., McCusker P., Timson D.J., Morphew R.M., Brophy P.M., Marks N.J., Mousley A., Maule A.G. (2016). Calmodulin disruption impacts growth and motility in juvenile liver fluke. Parasites Vectors.

[8] Mejia P., Diez-Silva M., Kamena F., Lu F., Fernandes S.M., Seeberger P.H., Davis A.E., Mitchell J.R. (2016). Human C1-Inhibitor Suppresses Malaria Parasite Invasion and Cytoadhesion via Binding to Parasite Glycosylphosphatidylinositol and Host Cell Receptors. J. Infect. Dis..

[9] Liu J., Pan T., You X., Xu Y., Liang J., Limpanont Y., Sun X., Okanurak K., Zheng H., Wu Z. (2015). SjCa8, a calcium-binding protein from *Schistosoma japonicum*, inhibits cell migration and suppresses nitric oxide release of RAW264.7 macrophages. Parasites Vectors.

[10] Feist S.W., Morris D.J., Alama-Bermejo G., Holzer A.S., Okamura B., Gruhl A., Bartholomew J. (2015). Cellular Processes in Myxozoans. Myxozoan Evolution, Ecology and Development.

[11] Gruhl A., Okamura B. (2012). Development and myogenesis of the vermiform *Buddenbrockia* (Myxozoa) and implications for cnidarian body plan evolution. EvoDevo.

[12] Alama-Bermejo G., Bron J.E., Raga J.A., Holzer A.S. (2012). 3D Morphology, Ultrastructure and Development of *Ceratomyxa puntazzi* Stages: First Insights into the Mechanisms of Motility and Budding in the Myxozoa. PLoS ONE.

[13] Noble E.R. (1941). Nuclear cycles in the life history of the protozoan genus *Ceratomyxa*. J. Morphol..

[14] Meglitsch P. (1960). Some coelozoic myxosporidia from New Zealand fishes I. General, and family Ceratomyxidae. Trans. Proc. R. Soc. N. Z..

[15] Sitjà-Bobadilla A., Palenzuela O., Alvarez-Pellitero P. (1995). *Ceratomyxa sparusaurati n*. sp. (Myxosporea: Bivalvulida), a new parasite from cultured gilthead seabream (*Sparus aurata* L.) (Teleostei: Sparidae): Light and electron microscopic description. J. Eukaryot. Microbiol..

[16] Cho J.B., Kwon S.R., Kim S.K., Nam Y.K., Kim K.H. (2004). Ultrastructure and development of *Ceratomyxa protopsettae* Fujita, 1923 (Myxosporea) in the gallbladder of cultured olive flounder, *Paralichthys olivaceus*. Acta Protozool..

[17] Hartigan A., Estensoro I., Vancová M., Bílý T., Patra S., Eszterbauer E., Holzer A.S. (2016). New cell motility model observed in parasitic cnidarian *Sphaerospora molnari* (Myxozoa:Myxosporea) blood stages in fish. Sci. Rep..

[18] Adriano E., Okamura B. (2017). Motility, morphology and phylogeny of the plasmodial worm, *Ceratomyxa vermiformis n*. sp. (Cnidaria: Myxozoa: Myxosporea). Parasitology.

[19] Bjork S.J., Bartholomew J.L. (2010). Invasion of *Ceratomyxa shasta* (Myxozoa) and comparison of migration to the intestine between susceptible and resistant fish hosts. Int. J. Parasitol..

[20] Bartholomew J.L., Whipple M.J., Stevens D.G., Fryer J.L. (1997). The Life Cycle of *Ceratomyxa shasta*, a Myxosporean Parasite of Salmonids, Requires a Freshwater Polychaete as an Alternate Host. J. Parasitol..

[21] Atkinson S.D., Bartholomew J.L. (2010). Disparate infection patterns of *Ceratomyxa shasta* (Myxozoa) in rainbow trout (*Oncorhynchus mykiss*) and Chinook salmon (*Oncorhynchus tshawytscha*) correlate with internal transcribed spacer-1 sequence variation in the parasite. Int. J. Parasitol..

[22] Atkinson S.D., Bartholomew J.L. (2010). Spatial, temporal and host factors structure the *Ceratomyxa shasta* (Myxozoa) population in the Klamath River basin. Infect. Genet. Evol..

[23] Hurst C.N., Bartholomew J.L. (2012). *Ceratomyxa shasta* genotypes cause differential mortality in their salmonid hosts. J. Fish Dis..

[24] Stinson M.E.T., Atkinson S.D., Bartholomew J.L. (2018). Widespread Distribution of *Ceratonova shasta* (Cnidaria: Myxosporea) Genotypes Indicates Evolutionary Adaptation to its Salmonid Fish Hosts. J. Parasitol..

[25] Ibarra A., Gall G., Hedrick R. (1991). Susceptibility of two strains of rainbow trout *Oncorhynchus mykiss* to experimentally induced infections with the myxosporean *Ceratomyxa Shasta*. Dis. Aquat. Org..

[26] Stocking R.W., Holt R.A., Foott J.S., Bartholomew J.L. (2006). Spatial and Temporal Occurrence of the Salmonid Parasite *Ceratomyxa shasta* in the Oregon–California Klamath River Basin. J. Aquat. Anim. Health.

[27] Hallett S., Bartholomew J. (2006). Application of a real-time PCR assay to detect and quantify the myxozoan parasite *Ceratomyxa shasta* in river water samples. Dis. Aquat. Org..

[28] Hallett S.L., Ray R.A., Hurst C.N., Holt R.A., Buckles G.R., Atkinson S.D., Bartholomew J.L. (2012). Density of the Waterborne Parasite *Ceratomyxa shasta* and Its Biological Effects on Salmon. Appl. Environ. Microbiol..

[29] Atkinson S.D., Hallett S.L., Bartholomew J.L. (2018). Genotyping of individual *Ceratonova shasta* (Cnidaria: Myxosporea) myxospores reveals intra-spore ITS-1 variation and invalidates the distinction of genotypes II and III. Parasitology.

[30] Ye J., Coulouris G., Zaretskaya I., Cutcutache I., Rozen S., Madden T.L. (2012). Primer-BLAST: A tool to design target-specific primers for polymerase chain reaction. BMC Bioinform..

[31] Schmittgen T.D., Livak K.J. (2008). Analyzing real-time PCR data by the comparative CT method. Nat. Protoc..

[32] Paluch E.K., Raz E. (2013). The role and regulation of blebs in cell migration. Curr. Opin. Cell Boil..

[33] Charras G., Paluch E. (2008). Blebs lead the way: How to migrate without lamellipodia. Nat. Rev. Mol. Cell Boil..

[34] Maugis B., Brugues J., Nassoy P., Guilĺen N., Sens P., Amblard F. (2010). Dynamic instability of the intracellular pressure drives bleb-based motility. J. Cell Sci..

[35] Charras G., Charras G. (2008). A short history of blebbing. J. Microsc..

[36] Johnson K.E. (1976). Circus movements and blebbing locomotion in dissociated embryonic cells of an amphibian, *Xenopus laevis*. J. Cell Sci..

[37] Olson E.C.E. (1996). Onset of Electrical Excitability during a Period of Circus Plasma Membrane Movements in Differentiating *Xenopus neurons*. J. Neurosci..

[38] Mattila P.K., Lappalainen P. (2008). Filopodia: Molecular architecture and cellular functions. Nat. Rev. Mol. Cell Boil..

[39] Paňková K., Rösel D., Novotný M., Brábek J. (2010). The molecular mechanisms of transition between mesenchymal and amoeboid invasiveness in tumor cells. Cell. Mol. Life Sci..

[40] Galbraith C.G., Yamada K., Galbraith J.A. (2007). Polymerizing Actin Fibers Position Integrins Primed to Probe for Adhesion Sites. Science.

[41] Diz-Muñoz A., Romanczuk P., Yu W., Bergert M., Ivanovitch K., Salbreux G., Heisenberg C.-P., Paluch E.K. (2016). Steering cell migration by alternating blebs and actin-rich protrusions. BMC Boil..

[42] Fackler O.T., Grosse R. (2008). Cell motility through plasma membrane blebbing. J. Cell Boil..

[43] Casadevall A., Pirofski L. (2001). Host-Pathogen Interactions: The Attributes of Virulence. J. Infect. Dis..

[44] Hurst C., Alexander J., Dolan B., Jia L., Bartholomew J. (2019). Outcome of within-host competition demonstrates that parasite virulence doesn’t equal success in a myxozoan model system. Int. J. Parasitol. Parasites Wildl..

[45] Bunnell T.M., Burbach B.J., Shimizu Y., Ervasti J.M. (2011). β-Actin specifically controls cell growth, migration, and the G-actin pool. Mol. Boil. Cell.

[46] Gandhi M., Goode B.L., Clemen C.S., Eichinger L., Rybakin V. (2008). Coronin: The Double-Edged Sword of Actin Dynamics. The Coronin Family of Proteins. Subcellular Biochemistry vol 48.

[47] Hou X., Katahira T., Ohashi K., Mizuno K., Sugiyama S., Nakamura H. (2013). Coactosin accelerates cell dynamism by promoting actin polymerization. Dev. Boil..

[48] Petrie R.J., Gavara N., Chadwick R.S., Yamada K.M. (2012). Nonpolarized signaling reveals two distinct modes of 3D cell migration. J. Cell Boil..

[49] Watanabe K., Petri W.A. (2015). Molecular biology research to benefit patients with *Entamoeba histolytica* infection. Mol. Microbiol..

[50] Rikitake Y., Takai Y., Jeon K.W. (2011). Chapter three—Directional Cell Migration: Regulation by Small G Proteins, Nectin-like Molecule-5, and Afadin. International Review of Cell and Molecular Biology.

[51] Ridley A.J. (2015). Rho GTPase signalling in cell migration. Curr. Opin. Cell Boil..

[52] Lo C.M., Buxton D.B., Chua G.C., Dembo M., Adelstein R.S., Wang Y.L. (2004). Nonmuscle myosin IIb is involved in the guidance of fibroblast migration. Mol. Boil. Cell.

[53] Fenix A.M., Burnette D.T. (2015). A small part of myosin IIB takes on a big role in cell polarity. J. Cell Boil..

[54] Tovy A., Hertz R., Siman-Tov R., Syan S., Faust D., Guillén N., Ankri S. (2011). Glucose Starvation Boosts *Entamoeba histolytica* Virulence. PLoS Negl. Trop. Dis..

[55] Lamb C.A., O’Byrne S., Keir M.E., Butcher E.C. (2018). Gut-Selective Integrin-Targeted Therapies for Inflammatory Bowel Disease. J. Crohn Colitis.

