# Supplementary material for: Myxozoan Adhesion and Virulence: Ceratonova shasta on the Move
Source: Microorganisms. 2019 Sep 26;7(10):397. doi: 10.3390/microorganisms7100397 (PMC6843538; doi:10.3390/microorganisms7100397)
Supplement: Supplementary file 1 [file microorganisms-07-00397-s001.zip › microorganisms-589551/Movies_legend.docx]

**Movie 1.** Genotype IIR *C. shasta* group of stages from ascites of rainbow trout showing active and profuse blebbing. Time-lapse series, every 7 seconds, elapsed time 10 min.

**Movie 2.** Genotype IIR *C. shasta* stages from ascites of rainbow trout with non-polarized blebbing, or circus movement, which consisted on a massive cell membrane detachment initiated as a regular hemispherical bleb that will propagate quickly and circumnavigate the stage body. Both stages contain two spores, the second one showing a common mutation of spores with three valves. Time elapsed 2 min 10 s.

**Movie 3.** Genotype IIR *C. shasta* stage from ascites of rainbow trout showing the moment were the stage expels one of the already formed spores. Time-lapse series, every 7 seconds, elapsed time 6 min 25 s.

**Movie 4.** Genotype IIR *C. shasta* stages from ascites of rainbow trout showing active blebbing, followed by complete destruction of the primary cell. Time-lapse series, every 3 seconds, time elapsed 8 min.

**Movie 5.** Genotype IIR *C. shasta* crawling stages from ascites of rainbow trout. Crawling stages showed a very active anterior pole and a static, rigid uropod with small filopodia at rear. These stages were able to change the leading edge position and start moving in a complete different direction (01:31 minute). 10 times real speed, time elapsed 18 min.

**Movie 6.** Genotype IIR *C. shasta* stage from ascites of rainbow trout switching between different motility-cell protrusions: blebbing to lamellipodia with filopodia.

**Movie 7.** Genotype I *C. shasta* stages from ascites of Chinook salmon showing all-in-one exploratory behavior on a combination of cell protrusions: blebs, lamellipodia and filopodia, with fully direction-oriented migration. Notice long filaments in the posterior end of the stage anchoring the parasite to the clog of host-parasite cells. Parasite stages are able to migrate, pushing and moving forward between the clog of cells using active blebbing. Time-lapse series, every 10 seconds, time elapsed 6 min.

**Movie 8.** Genotype I *C. shasta* stage from ascites of Chinook salmon showing all-in-one motility. Notice the ability of moving forward and backward with this body configuration *i.e.* anterior active pole protruding blebs, lamellipodia and filopodia and posterior pole with long anchoring filaments. Time-lapse series, every 10 seconds, time elapsed 22 min.
