# Supplementary material for: Myxozoan Adhesion and Virulence: Ceratonova shasta on the Move
Source: Microorganisms. 2019 Sep 26;7(10):397. doi: 10.3390/microorganisms7100397 (PMC6843538; doi:10.3390/microorganisms7100397)
Supplement: Supplementary file 1 [file microorganisms-07-00397-s001.zip › microorganisms-589551/Supp_tables_.docx]

**Table S1.-** *C. shasta* motility genes and reference genes used in this study.

| **Gene name** | **Assembled contig number or GenBank Acc. No.** | **Length (bp)** | **Primer name** | **Primer sequence 5’-3’** | **Amplicon size (bp)** | **PE/CV (%)** | **Reference** |
| --- | --- | --- | --- | --- | --- | --- | --- |
| β-actin | TRINITY_DN23296_c1_g4_i1 | 459 | DN23296F | GTCGGCAATTCCTGGGTACA | 149 | 103.4 | Present study |
|  |  |  | DN23296R | TCCAACCGGCATTTTTAGGA |  |  |  |
| Coactosin | TRINITY_DN20273_c2_g1_i1 | 740 | DN20273F | GAACTCCTTCGCCAACCCAT | 141 | 95.4 | Present study |
|  |  |  | DN20273R | AGACGGGTGATGACTATAGCG |  |  |  |
| Coronin | TRINITY_DN21518_c3_g1_i1 | 1579 | DN21518F | TGACCCATTTAACGCAAACAGG | 145 | 102.3 | Present study |
|  |  |  | DN21518R | GGCCTGAACAGCTCTTCTCT |  |  |  |
| Myosin-10 | TRINITY_DN31226_c98_g1_i1 | 5903 | DN31226F | CCAAGCCAAACTTTGTCCGT | 140 | 104.3 | Present study |
|  |  |  | DN31226R | GTTGGGATAACCTTGACGGC |  |  |  |
| Integrin-β | TRINITY_DN28283_c11_g1_i2 | 2202 | DN28283F | TACTTCCTTCTGCGTGTGGC | 117 | 91.2 | Present study |
|  |  |  | DN28283R | ATGTTTTTGGAGCCATTGCACA |  |  |  |
| Talin | TRINITY_DN29288_c9_g1_i1 | 7462 | DN29288F | CCGCCCATCTTTTAACAGCC | 107 | 106.9 | Present study |
|  |  |  | DN29288R | AGGCCCTGTGTTGTTTGGAA |  |  |  |
| Rac1 | TRINITY_DN28282_c10_g1_i2 | 1427 | DN28282F | CGAGCTGGAAGATACAAGCGA | 143 | 109.7 | Present study |
|  |  |  | DN28282R | CCAGTGTCATGGTTGACGGA |  |  |  |
| RhoA | TRINITY_DN30297_c1_g1_i1 | 1002 | DN30297F | GGATACCGCAGGACAAGAGG | 141 | 107.2 | Present study |
|  |  |  | DN30297R | TGTCTTACTTCTGGAACCCACT |  |  |  |
| GAPDH | TRINITY_DN29264_c10_g2_i1 | 1930 | DN29264F | TGGGGCTAAACAGTTGGTGG | 152 | 103.2/1 | Present study |
|  |  |  | DN29264R | GTGGACATTTGAAAGGAGGCG |  |  |  |
| NADH | TRINITY_DN5700_c0_g1_i1 | 1492 | DN5700F | TTCCTTGAGTTCTGCCCAAGT | 154 | 102.4/0.8 | Present study |
|  |  |  | DN5700R | GCTGGTTACATACGCCCAGG |  |  |  |
| HPRT | TRINITY_DN5718_c0_g1_i1 | 935 | DN5718F | ACTTTCGATGATTGAAGAATTGGT | 140 | 96.5/1.1 | Present study |
|  |  |  | DN5718R | TGCTTAAAACTGTAATGGAAGCAGG |  |  |  |
| **Other reference genes tested in this study** | | | | | | **CV (%)** |  |
| SSU rDNA | AF001579 | 1643 | Cs1034F | CCAGCTTGAGATTAGCTCGGTAA | 71 | 4.7 | Hallett & Bartholomew 2006 |
|  |  |  | Cs1104R | CCCCGGAACCCGAAAG |  |  |  |
| LSU rDNA | FJ981818 | 3216 | CsLSUf1 | CTCGAAGTGATTCTGACG | 118 | 6.0 | Present study |
|  |  |  | CsLSUr1 | GGATCGCCGAGAATTT |  |  |  |
| EF-2 | KM392431 | 779 | CsEF2f1 | CTTCAGGCATAGGATGAG | 156 | 8.2 | Present study |
|  |  |  | CsEF2r1 | GCATGATCCTCCTCTAAAT |  |  |  |
| DNA-directed RNA polymerase II | TRINITY_DN29346_c0_g1_i1 | 923 | DN29346F | TGGAGGTTGAAGGTACGTGT | 156 | 1.3 | Present study |
|  |  |  | DN29346R | TCTGCCCCTTTATAGGACGA |  |  |  |
| Ornithine aminotransferase | TRINITY_DN31715_c0_g1_i1 | 1374 | CsOrnAmf1 | CAACCAAGGACATTGTCA | 149 | 6.8 | Present study |
|  |  |  | CsOrnAmr1 | GCTATGATAAGGCTTTGC |  |  |  |

**Table S2.-** Parasite copy numbers ± SD (standard deviation) and range in parenthesis in gills, blood and intestine infected with type 0 and IIR at different time points during the experimental infections (n=5 fish per each genotype and sampling time point). Parasite fold difference between genotypes (IIR:0).

Gills

| Day p.e. | Genotype 0 copies | Genotype IIR copies | Fold |
| --- | --- | --- | --- |
| 1 | 4 ± 2 (1-7) | 3 ± 0.4 (2-3) | 0.8 |
| 15 | 35 ± 12 (20-52) | 33 ± 9 (22-46) | 0.9 |
| 29 | 31 ± 20 (14-63) | 13,922 ± 13,422 (162-32,166) | 443 |

Blood

| Day p.e. | Genotype 0 copies | Genotype IIR copies | Fold |
| --- | --- | --- | --- |
| 1 | 3 ± 4 (0-10) | 1 ± 1 (0-2) | 0.3 |
| 15 | 1 ± 1 (0-2) | 150 ± 335 (0 -750) | 284 |
| 29 | 1 ± 1 (1-2) | 10,374 ± 19,728 (0-45,355)* | - |

* Large variability

Intestine

| Day p.e. | Genotype 0 copies | Genotype IIR copies | Fold |
| --- | --- | --- | --- |
| 1 | 0-0.2 | 0-0.2 | - |
| 7 | 559 ± 346 (340-1,166) | 13,621 ± 7,141 (8,124-23,948) | 24 |
| 15 | 4,984 ± 2,433 (1,390-7,066) | 254,351 ± 152,954 (100,517-511,126) | 51 |
| 22 | 20,678 ± 12,210 (9,001-36,949) | 3,143,538 ± 912,338 (1,824,591-4,202,016) | 152 |
| 29 | 15,681 ± 5,788 (6,743-20,609) | 2,048,415 ± 1,135,623 (556,851-3,486,744) | 131 |
| 60 | 1,758 ± 1,613 (273-4,479) | - | - |

**Table S3.-** Motility genes fold change mean ± SD calculated using 2^-ddCq^ method per gene and sampling day post exposure (dpe) between *C. shasta* genotypes IIR and 0 in the intestine (n=5 fish per each genotype and sampling time point). Statistical differences between mean fold changes were calculated using Tukey´s method for multiple comparison after one-way ANOVA between the four sampling time points (7, 15, 22, 29 p.e.).

| **Gene** | **7 dpe** | **15 dpe** | **22 dpe** | **29 dpe** | **Statistical results** |
| --- | --- | --- | --- | --- | --- |
| β-actin | 21.03±1.8 | 12.39±0.83 | 4.14±0.53 | 4.3±0.42 | F=58.977; df=3; p<0.001 |
| Coactosin | 0.43 ±0.03 | 0.46±0.06 | 0.31±0.03 | 0.39±0.02 | F=2.767; df=3; p=0.076 |
| Coronin | 0.38±0.02 | 0.26±0.02 | 0.52±0.02 | 0.36±0.03 | F=19.178; df=3; p<0.001 |
| Myosin-10 | 0.28±0.04 | 0.14±0.01 | 0.38±0.05 | 0.42±0.06 | F=7.853; df=3; p=0.002 |
| Integrin-β | 0.06±0.02 | 0.82±0.63 | 30.96±5.21 | 54.3±9.38 | H=15.114; df=3; p=0.002 |
| Talin | 0.11±0.03 | 0.25±0.16 | 1.62±0.46 | 7.33±0.45 | H=15.274; df=3; p=0.002 |
| RhoA | 1.35±0.27 | 2.29±0.32 | 1.67±0.16 | 2.46±0.25 | F=4.135; df=3; p=0.024 |
| Rac1 | 0.27±0.02 | 0.51±0.05 | 0.81±0.05 | 0.7±0.05 | F=28.937; df=3; p<0.001 |

**Table S4.-** Motility genes relative change mean ± SD using 2^-dCq^ method per gene, sampling day per each genotype (0 and IIR) against their respective reference genes in the intestine (n=5 fish per each genotype and sampling time point). Statistical differences between mean relative change were calculated using Tukey´s method for multiple comparison after one-way ANOVA between the four sampling time points (7, 15, 22, 29 p.e.) and for each genotype.

| **Gene** | **Genotype** | **7 dpe** | **15 dpe** | **22 dpe** | **29 dpe** | **Statistical results** |
| --- | --- | --- | --- | --- | --- | --- |
| β-actin | 0 | 2.7 ± 1.6 | 7.7 ± 3.7 | 9.6 ± 2.6 | 5.9 ± 3.3 | F=4.192; df=3; p=0.024 |
|  | IIR | 73.2 ± 14.0 | 80.4 ± 12.0 | 37.5 ± 10.8 | 28.2 ± 6.2 | F=27.012; df=3; p<0.001 |
| Coactosin | 0 | 4.1 ± 2.9 | 6.7 ± 2.3 | 8.1 ± 3.0 | 7.6 ± 3.9 | F=1.565; df=3; p=0.239 |
|  | IIR | 1.8 ± 0.3 | 2.9 ± 0.8 | 2.3 ± 0.5 | 2.1 ± 0.3 | F=3.731; df=3; p=0.033 |
| Coronin | 0 | 3.1 ± 2.1 | 3.2 ± 0.4 | 2.9 ± 0.8 | 3.0 ± 1.8 | F=0.0342; df=3; p=0.991 |
|  | IIR | 1.3 ± 0.2 | 1.0 ± 0.2 | 1.4 ± 0.1 | 1.3 ± 0.2 | F=3.584; df=3; p=0.037 |
| Myosin-10 | 0 | 9.5 ± 5.5 | 6.5 ± 1.8 | 3.9 ± 1.4 | 2.4 ± 2.1 | H=8.966; df=3; p=0.030 |
|  | IIR | 1.4 ± 0.5 | 0.9 ± 0.2 | 1.3 ± 0.4 | 1.0 ± 0.3 | H=5.880; df=3; p=0.118 |
| Integrin-β | 0 | 0.15 ± 0.08 | 0.06 ± 0.02 | 0.01 ± 0.01 | 0.01 ± 0.01 | H=15.000; df=3; p=0.002 |
|  | IIR | 0.01 ± 0.0 | 0.06 ± 0.12 | 0.38 ± 0.17 | 0.55 ± 0.29 | F=10.595; df=3; p<0.001 |
| Talin | 0 | 0.23 ± 0.25 | 0.05 ± 0.02 | 0.03 ± 0.01 | 0.03 ± 0.04 | H=11.236; df=3; p=0.011 |
|  | IIR | 0.01 ± 0.01 | 0.01 ± 0.02 | 0.04 ± 0.02 | 0.05 ± 0.03 | H=8.246; df=3; p=0.041 |
| RhoA | 0 | 2.0 ± 0.8 | 1.3 ± 0.2 | 1.9 ± 1.9 | 1.1 ± 0.6 | H=3.537; df=3; p=0.316 |
|  | IIR | 2.8 ± 2.0 | 2.9 ± 0.9 | 2.0 ± 0.4 | 2.4 ± 0.7 | H=2.909; df=3; p=0.406 |
| Rac1 | 0 | 1.3 ± 0.7 | 1.1 ± 0.2 | 1.2 ± 0.3 | 1.1 ± 0.6 | F=0.152; df=3; p=0.927 |
|  | IIR | 0.4 ± 0.1 | 0.6 ± 0.1 | 0.9 ± 0.1 | 0.9 ± 0.2 | F=25.359; df=3; p<0.001 |

**Table S5**.- Motility and migration features of different genotypes of *Ceratonova shasta* infecting rainbow trout *Oncorhynchus mykiss*.

| ***C. shasta* genotype** | **IIR** | **0** |
| --- | --- | --- |
| **Host** | Allopatric *O. mykiss* | |
| **Type of infection** | Systemic, enteronecrosis | Chronic, asymptomatic |
| **Ascites production** | Yes | No |
| **Mortality** | High, frequently lethal, up to 100% | Low or absent |
| **Proliferation** | Extremely high, ↑ β-actin, fast replication | Low, ↓ β-actin, slow replication |
| **Spore production** | Fast, before host death  (weeks) | Delayed, release for long periods of time  (years) |
| **Parasite feeding needs** | High | Low |
| **Parasite invasion strategy** | Continuous invasion  (proliferating in blood/gills) | Initial invasion  (no proliferation in blood/gills) |
| **Dispersion/Spreading** | High | Low |
| **Cell protrusions types** | Blebs, lamellipodia, filopodia | NA - assumed similar capacities |
| **Cell protrusion plasticity** | High | NA - Probably not required |
| **Early promoted motility** | Amoeboid motility - ↑RhoA | Mesenchymal motility –adhesion ↑ Integrin-β, talin |
| **Main type of protrusion in early infection** | Blebs | Lamellipodia and filopodia (hypothetical) |
| **Late promoted motility** | Mesenchymal motility – high adhesion - ↑ Integrin-β, talin, Rac1 | Probably very low motility needs, remains in the ECM gaps |
| **Main type of protrusion in late infection** | Lamellipodia and filopodia | - |
| **Parasite adhesion** | High late - ↑ Integrin-β, talin | High early - ↑ Integrin-β, talin |
| **Remodeling and destruction of the ECM** | Extremely high | Low |
| **Immune response** | Local and acute, high in pro-inflammatory cytokines | Local and chronic |
